# Prevalence and risk factors of *Helicobacter pylori* infection in Korea: Nationwide multicenter study over 13 years

**DOI:** 10.1186/1471-230X-13-104

**Published:** 2013-06-24

**Authors:** Seon Hee Lim, Jin-Won Kwon, Nayoung Kim, Gwang Ha Kim, Jung Mook Kang, Min Jung Park, Jeong Yoon Yim, Heung Up Kim, Gwang Ho Baik, Geom Seog Seo, Jeong Eun Shin, Young-Eun Joo, Joo Sung Kim, Hyun Chae Jung

**Affiliations:** 1 Seoul National University Hospital, Healthcare System Gangnam Center, Healthcare Research Institute, Seoul, Korea; 2 College of Pharmacy, Kyungpook National University, Daegu, Korea; 3 Department of Internal Medicine, Seoul National University Bundang Hospital, Seongnam, Korea; 4 Department of Internal Medicine and Liver Research Institute, Seoul National University College of Medicine, Seoul, Korea; 5 Department of Internal Medicine, Pusan National University School of Medicine, Busan, Korea; 6 Department of Internal Medicine, School of Medicine, Jeju National University, Jeju, Korea; 7 Department of Internal Medicine, Hallym University College of Medicine, Chuncheon, Korea; 8 Department of Internal Medicine, Wonkwang University Hospital, Iksan, Korea; 9 Department of Internal medicine, Dankook University College of Medicine, Chonan, Korea; 10 Department of Internal Medicine, Chonnam National University Medical School, Gwangju, Korea

**Keywords:** Helicobacter pylori, Seroprevalence, Epidemiology, Cohort

## Abstract

**Background:**

The aim of this study was to evaluate the time trend of seropositivity of *Helicobacter pylori* (*H*. *pylori*) over the period of 13 years in an asymptomatic Korean population, and investigate associated risk factors.

**Methods:**

This cross-sectional nationwide multicentre study surveyed anti-*H*. *pylori* IgG antibodies in 19,272 health check-up subjects (aged [greater than and equal to]16 years) in 2011. Risk factors for *H*. *pylori* infection were investigated using logistic regression. Seropositivity in asymptomatic subjects without *H*. *pylori* eradication was compared between the years 1998 and 2005. Birth cohort effects were also evaluated.

**Results:**

After exclusion of subjects with a history of *H*. *pylori* eradication therapy (n = 3,712, 19.3%) and gastric symptoms (n = 4,764, 24.7%), the seroprevalence of *H*. *pylori* infection was 54.4% in 10,796 subjects. This was significantly lower than the seroprevalence of 59.6% in 2005 and that of 66.9% in 1998, and this decrease of seropositivity of *H*. *pylori* became widespread across all ages and in most areas of the country. This decreasing trend could be explained by cohort analysis. All younger birth cohorts had a lower seroprevalence of *H*. *pylori* than older birth cohorts at the same age. Decreased seroprevalence within the same birth cohorts also accounted for this phenomenon. Clinical risk factors of *H*. *pylori* infection were higher cholesterol level ([greater than and equal to] 240 mg/dl) (OR = 1.33; 95% CI = 1.14-1.54), male gender, older age, low income, and residence in a rural area.

**Conclusions:**

A decreasing trend of *H*. *pylori* seroprevalence due to a birth cohort effect requires further studies on its related human host factors as well as socio-economic and hygienic factors. In addition, the relationship between *H*. *pylori* infection and high cholesterol level needs more investigation regarding underlying pathogenesis.

## Background

*Helicobacter pylori* (*H*. *pylori*), a cause of peptic ulcer disease, gastric adenocarcinoma, and low-grade gastric mucosa associated lymphoid tissue (MALT) lymphoma [1] has been falling due to improved sanitation and better living conditions [2,3]. However, its prevalence is reported to be still high, especially in Asia including South Korea. From the public health perspective, observation of prevalence trends and confirmation of risk factors for *H*. *pylori* infection are important to establish health policies to prevent *H*. *pylori* related diseases.

There are many studies regarding the prevalence and risk factors of *H*. *pylori* infection, and older age was commonly considered as the main risk factor [4,5]. One study mentioned that adults have a continuous risk of *H*. *pylori* infection, resulting in increased seroprevalence during lifetime as a function of age [6]. However, this does not mean that young people have a higher seroprevalence when they get older, showing that cross sectional presentation does not necessarily give an accurate picture of lifetime trends. Also, there are limited studies on lifetime trends for *H*. *pylori* seroprevalence [7,8].

In South Korea, previous study also indicated a decreasing pattern of *H*. *pylori* infection during a time period between 1998 and 2005 [9]. As Korea is in a dynamic state of progression from a developing country into a developed country, it may be valuable to evaluate the seroprevalence of *H*. *pylori* in Korea. In accordance with this point of view, the aim of this study was to investigate the trends of seropositivity of *H*. *pylori* in asymptomatic Korean subjects over 16 years of age together with cohort effects between the years 1998 and 2011, and to find factors related to *H*. *pylori* infection.

## Methods

### Study population

This is a cross-sectional nationwide multicentre study of adult subjects aged 16 years or older who visited healthcare centers for routine health check-up between January and December 2011 in South Korea. The subjects were enrolled prospectively in 2011 under a predefined protocol. The institutions participating in this study were healthcare centers located in Seoul and in the seven provinces of South Korea.

Informed consent was obtained from each subject. All subjects were invited to answer the questionnaire which was the same as previous study’s [9] under the supervision of a well-trained interviewer. The questionnaire included information regarding demographic data (i.e. age, sex, and residence), socioeconomic data (i.e. monthly income and education level), medical history (such as *H*. *pylori* eradication therapy, history of gastric operation, and family history of gastric cancer (GC)), and upper gastrointestinal (GI) symptoms (such as indigestion, bloating, epigastric soreness, regurgitation, or heartburn), that persisted for at least one month within the last 3 years.

Subjects were categorized into 3 education levels: low (middle school graduate or less), middle (high school graduate or university dropout), and high (university graduate or graduate of a postgraduate course). Monthly family income was classed as 3 groups: low household income (< US $ 3,000 per month), middle income (US $ 3,000 to 10,000 per month), and high income (> US $ 10,000 per month).

### Clinical and laboratory evaluations

Anthropometric measurements (weight and height) were done by trained nurses using a standardized protocol.

Blood samples were obtained from the antecubital vein in the morning after overnight fasting, and serum samples were separated after centrifugation. Serum cholesterol, triglyceride, and fasting glucose were measured by an automatic analyser, Alisei® (Seac, Pomezia, Italy). To compare these results according to seropositivity of *H*. *Pylori*, we categorized the level of total cholesterol (TC) as normal (≤240 mg/dl) and abnormal (>240 mg/dl), trigryceride (TG) as ≤150 mg/dl and >150 mg/dl, and fasting glucose as ≤100 mg/dl and >100 mg/dl, respectively.

Anti-*H*. *pylori* IgG was measured using *H*. *pylori*-EIA-Well in Healthcare System Gangnam Center and Genedia *H*. *pylori* ELISA at the remaining centers using the same kits as those in the previous studies [9,10]. Genedia *H*. *pylori* ELISA, developed from Korean *H*. *pylori* strains showed a sensitivity of 97.8% and a specificity of 92% [11]. *H*. *pylori*-EIA-Well showed a sensitivity of 95.6% and a specificity of 97.8% when Genedia *H*. *pylori* ELISA was used as the gold standard [9].

### Statistical analysis

#### Evaluation of risk factors of each group according to eradication of H. pylori

Demographic and clinical information were summarized by descriptive statistics. To investigate risk factors for *H*. *pylori* seropositivity and influential factors having a history of *H*. *pylori* eradication, multivariable logistic regression was used. A significance level of *p* < 0.05 was used for all analyses.

#### Comparison of trends of seroprevalence of H. pylori in 1998, 2005, and 2011

Trends of seroprevalence of *H*. *pylori* were compared using the published data of 1998 [10] and 2005 [9]. For this comparison, study subjects in each time period were restricted to asymptomatic subjects without a history of *H*. *pylori* eradication and gastric operation. For statistical comparison of trends of seroprevalence of *H*. *pylori* in 1998, 2005, and 2011, the Cochrane-Armitage trend test, which is a modified Pearson chi-square test to examine the association between a binary outcome and a variable with multiple categories with order, was conducted.

#### Analysis of cohort effects

In addition, the seroprevalence of *H*. *pylori* by birth cohort group was also drawn. To examine birth cohort effects, we created synthetic cohorts from the successive cross-sectional data of 1998, 2005, and 2011. For this analysis, relevant raw data in 1998 and 2005 were obtained from the authors and reconstructed for the analysis of birth cohort. Data from 1998 was considered to be those in 1999 because the successive cross-sectional data should span with same interval. The interval of three cross-sectional data was 6 years. The aggregate birth cohort from 1930 to 1972 was restructured into 8 groups using the standard approach for cohort analysis [12]. In detail, a birth cohort was obtained by subtracting age from year (i.e. Birth cohort of 1974.5 (birth cohort of 1972–77) = Year of 1998 – Age of 24.5 (22–27 years old)). For example, people aged 22–27 years in 1998 (considered as data in 1999), those aged 28–33 years in 2005, and those aged 34–39 years in 2011 were considered to be in the same birth cohort, born between 1972 and 1977. Using this approach for other age groups in each year, eight birth cohorts (1972–77, 1966–71, 1960–65, 1954–59, 1948–53, 1942–47,1936-41,1930-35) had three estimates of *H*. *pylori* seroprevalence at 6 year intervals for 12 years.

### Ethics statement

The protocol of this study was approved by the main Institutional Review Board of Seoul National University Hospital (IRB No. H-1011-038-339).

## Results

### Seroprevalence and eradication history of *H*. *pylori* in total subjects

**Figure 1:**
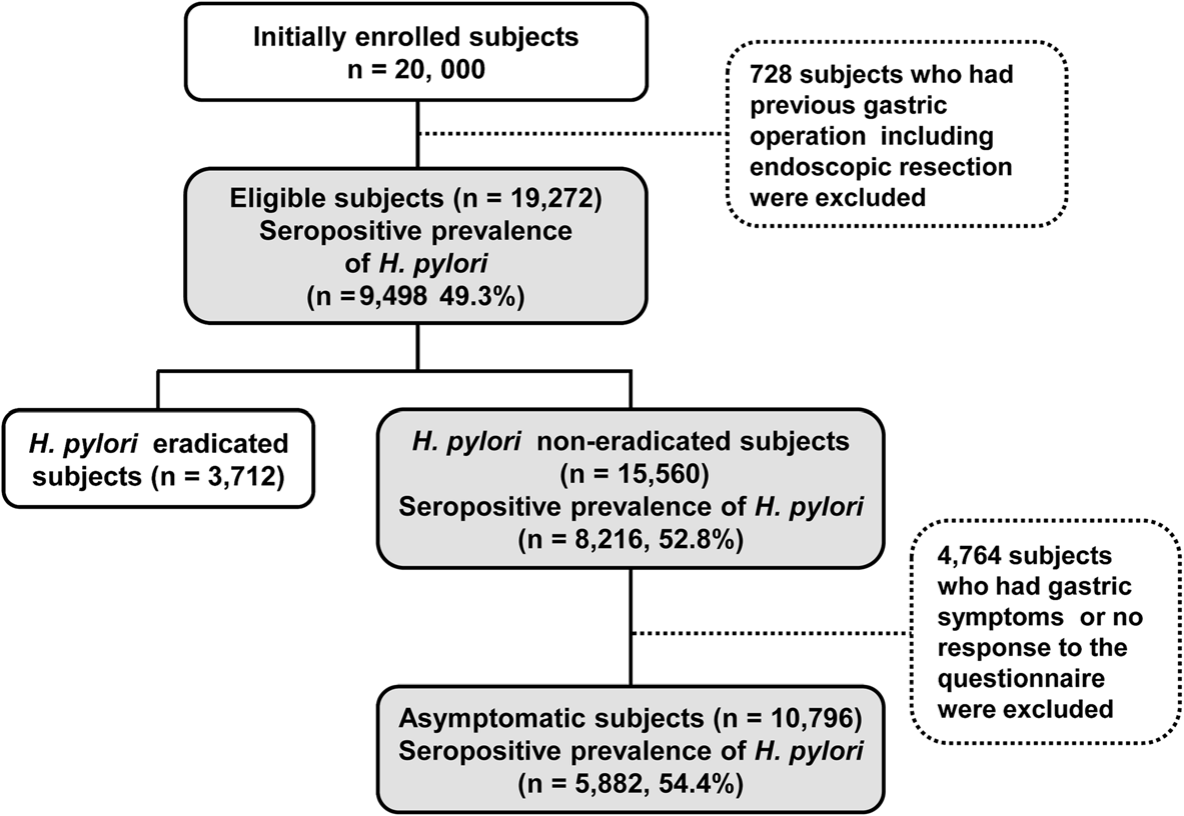
Study flowchart in 2011.

The seroprevalence of *H*. *pylori* was 52.8% (8,216/15,560) after exclusion of *H*. *pylori* eradicated history in 19,272 eligible subjects and 54.4% (5,882/10,796) after exclusion of symptomatic subjects (Figure 1).

The demographics and clinical characteristics by group are presented in Table 1.

**Table 1:** Baseline characteristics of subjects

		**Subjects without GI operation history**		**Subjects without history of *H. pylori *eradication**		**Asymptomatic subjects without history of *H***. ***pylori *eradication**	
		**n**	**(%)**	**n**	**(%)**	**n**	**(%)**
Total		19,272	(100.0)	15,560	(100.0)	10,796	(100.0)
Sex	Male	10,557	(54.8)	8,311	(53.4)	6,085	(56.4)
	Female	8,715	(45.2)	7,249	(46.6)	4,711	(43.6)
	Subtotal	19,272	(100.0)	15,560	(100.0)	10,796	(100.0)
Age (years)	16-19	34	(0.2)	33	(0.2)	17	(0.2)
	20-29	798	(4.1)	777	(5.0)	421	(3.9)
	30-39	2,853	(14.8)	2,607	(16.8)	1,659	(15.4)
	40-49	5,087	(26.4)	4,198	(27.0)	2,913	(27.0)
	50-59	6,176	(32.0)	4,709	(30.3)	3,403	(31.5)
	60-69	3,358	(17.4)	2,493	(16.0)	1,840	(17.0)
	≥70	966	(5.0)	743	(4.8)	543	(5.0)
	Subtotal	19,272	(100.0)	15,560	(100.0)	10,796	(100.0)
Geographic area	Seoul	10,755	(55.8)	8,515	(54.8)	5,829	(54.0)
	Gyeonggi	3,025	(15.7)	2,403	(15.5)	1,683	(15.6)
	Chungcheong	863	(4.5)	740	(4.8)	536	(5.0)
	Kyungsang	1,630	(8.5)	1,313	(8.4)	914	(8.5)
	Cholla	1,807	(9.4)	1,553	(10.0)	1,194	(11.1)
	Kangwon	588	(3.1)	466	(3.0)	331	(3.1)
	Jeju	589	(3.1)	555	(3.6)	299	(2.8)
	Subtotal^*^	19,257	(100.0)	15,545	(100.0)	10,786	(100.0)
Household income^**^	low	2,114	(12.5)	1,838	(13.5)	1,247	(13.2)
	medium	11,049	(65.2)	8,843	(65.1)	6,197	(65.6)
	high	3,781	(22.3)	2,910	(21.4)	2,005	(21.2)
	Subtotal^*^	16,944	(100.0)	13,591	(100.0)	9,449	(100.0)
Education level^***^	low	1,743	(9.5)	1,458	(9.9)	984	(9.7)
	medium	3,667	(20.0)	3,004	(20.4)	1,981	(19.4)
	high	12,928	(70.5)	10,250	(69.7)	7,223	(70.9)
	Subtotal*	18,338	(100.0)	14,712	(100.0)	10,188	(100.0)
Body mass index (kg/m^2^)	<18.5	840	(4.4)	736	(4.8)	412	(3.9)
	18.5 - <23.0	7,696	(40.6)	6,245	(40.7)	4,237	(39.8)
	23.0 - <25.0	4,818	(25.4)	3,821	(24.9)	2,759	(25.9)
	≥25.0	5,625	(29.6)	4,530	(29.5)	3,249	(30.5)
	Subtotal^*^	18,979	(100.0)	15,332	(100.0)	10,657	(100.0)
Cholesterol (mg/dl)	<240	16,671	(90.5)	13,417	(90.4)	9,228	(90.1)
	≥240	1,755	(9.5)	1,428	(9.6)	1,013	(9.9)
	Subtotal^*^	18,426	(100.0)	14,845	(100.0)	10,241	(100.0)
TG (mg/dl)	<150	14,651	(79.7)	11,873	(80.1)	8,118	(79.4)
	≥150	3,736	(20.3)	2,941	(19.9)	2,102	(20.6)
	Subtotal*	18,387	(100.0)	14,814	(100.0)	10,220	(100.0)
Glucose (mg/dl)	<100	12,999	(70.7)	10,667	(72.0)	7,206	(70.5)
	100 - <126	4,438	(24.1)	3,399	(22.9)	2,459	(24.1)
	≥126	955	(5.2)	747	(5.0)	555	(5.4)
	Subtotal*	18,392	(100.0)	14,813	(100.0)	10,220	(100.0)
Family history of gastric cancer	No	16,470	(86.6)	13,418	(87.5)	9,473	(88.2)
	Yes	2,556	(13.4)	1,910	(12.5)	1,270	(11.8)
	Subtotal*	19,026	(100.0)	15,328	(100.0)	10,743	(100.0)
GI symptoms	No	13,121	(68.8)	10,796	(70.3)		
	Yes	5,954	(31.2)	4,568	(29.7)		
	Subtotal*	19,075	(100.0)	15,364	(100.0)		

^*^Subjects with missing values were excluded.

^**^Household income was classified as low (less than US $ 3,000), medium (US $ 3,000 to 10,000), or high (more than US $ 10,000).

^***^Education level was classified as low (middle school graduates or less), middle (high school graduates or university dropouts), or high (university graduates or graduates of a postgraduate course).

*GI* gastrointestinal.

Among the 19,272 subjects, 19.3% reported a history of eradication therapy for *H*. *pylori* infection. By logistic regression modeling, the influencing factors for having a history of eradication therapy for *H*. *pylori* infection were male, older age, higher income, living in Seoul (Capital area), the presence of GI symptom and GC family history (Table 2).

**Table 2:** **Multivariate analysis of factors affecting *H***. ***pylori *eradication therapy**

		**Total**	**Subjects with history of *H. pylori *eradication**		**Odds ratio**	**95% CI**	
			**n**	**%**			
Total		19,272	3,712	19.3			
Sex	Male	10,557	2,246	21.3	Ref		
	Female	8,715	1,466	16.8	0.77	0.71	0.84
Age (years)	16-19	34	1	2.9	2.27	0.28	18.21
	20-29	798	21	2.6	Ref		
	30-39	2,853	246	8.6	4.22	2.44	7.31
	40-49	5,087	889	17.5	9.32	5.44	15.94
	50-59	6,176	1,467	23.8	14.33	8.39	24.48
	60-69	3,358	865	25.8	16.91	9.87	28.97
	≥70	966	223	23.1	16.73	9.58	29.20
Geographic area	Seoul	10,755	2,240	20.8	Ref		
	Gyeonggi	3,025	622	20.6	0.97	0.87	1.08
	Chungcheong	863	123	14.3	0.76	0.61	0.94
	Kyungsang	1,630	317	19.4	0.85	0.74	0.99
	Cholla	1,807	254	14.1	0.68	0.58	0.80
	Kangwon	588	122	20.7	1.30	1.04	1.64
	Jeju	589	34	5.8	0.27	0.19	0.39
Household income*	Low	2,114	276	13.1	0.70	0.58	0.83
	Medium	11,049	2,206	20.0	0.96	0.87	1.05
	High	3,781	871	23.0	Ref		
Education level**	Low	1,743	285	16.4	0.70	0.59	0.84
	Medium	3,667	663	18.1	0.87	0.78	0.97
	High	12,928	2,678	20.7	Ref		
GI symptoms	No	13,121	2,325	17.7	Ref		
	Yes	5,954	1,386	23.3	1.60	1.47	1.73
Family history of gastric cancer	No	16,470	3,052	18.5	Ref		
	Yes	2,556	646	25.3	1.34	1.21	1.49

*, **, GI, same as those of Table 1.

*CI* confidence Interval, *Ref* reference.

Total subject number of multivariable logistic regression was 16,770.

### Risk factors for *H*. *pylori* infection in asymptomatic subjects without a history of *H*. *pylori* eradication

The risk factors for *H*. *pylori* infection in asymptomatic subjects without a history of *H*. *pylori* eradication were significantly associated with gender, age, geographic area, economic status, education level, and cholesterol level (Table 3). Seropositivity of *H*. *pylori* was significantly lower in females than in males (OR = 0.79, 95% CI = 0.71-0.87). By age, seroprevalence increased in a nearly linear fashion from 20 to 59 years of age. However, the prevalence remained steady from 60 years of age. Regarding residence, when compared with Seoul, other provinces except for Gyeonggi and Kangwon had a higher risks of *H*. *pylori* seropositivity. Subjects with high income and high education level had a lower likelihoods of having *H*. *pylori* seropositivity. Subjects with a higher TC level (≥240 mg/dl) had a 30% higher likelihood of having *H*. *pylori* seropositivity compared with subjects with a lower TC (<240 mg/dl) (OR = 1.33, 95% CI = 1.14-1.54) after adjustments for BMI, age, and income level. However, blood glucose level and TG level did not affect the seropositivity of *H*. *pylori* infection after adjustment for other variables. Family history of GC and BMI level did not affect the seropositivity of *H*. *pylori*.

**Table 3:** **Risk factors for *H***. ***pylori *seropositivity in asymptomatic subjects without a history of *H***. ***pylori *eradication and gastric operation (Multivariable logistic regression)**

		**Total**	***H***. ***pylori *Seropositivity**				
			**N**	**%**	**Odds ratio**	**95% CI**	
Total		10,796	5,882	54.5			
Sex	Male	6,085	3,472	57.1	Ref		
	Female	4,711	2,410	51.2	0.79	0.71	0.87
Age (years)	16-19	17	2	11.8	0.55	0.11	2.68
	20-29	421	111	26.4	Ref		
	30-39	1,659	698	42.1	1.55	1.18	2.04
	40-49	2,913	1,531	52.6	2.39	1.83	3.11
	50-59	3,403	2,088	61.4	3.52	2.70	4.60
	60-69	1,840	1,134	61.6	3.57	2.70	4.71
	≥70	543	318	58.6	3.11	2.24	4.31
Geographic area	Seoul	5,829	2,917	50.0	Ref		
	Gyeonggi	1,683	898	53.4	1.07	0.95	1.21
	Chungcheong	536	297	55.4	1.29	1.05	1.58
	Kyungsang	914	595	65.1	1.29	1.06	1.57
	Cholla	1,194	790	66.2	1.66	1.41	1.96
	Kangwon	331	200	60.4	1.19	0.92	1.54
	Jeju	299	176	58.9	1.36	1.05	1.77
Household Income*	Low	1,247	785	63.0	1.21	1.00	1.45
	Medium	6,197	3,416	55.1	1.07	0.96	1.19
	High	2,005	1,042	52.0	Ref		
Education**	Low	984	629	63.9	1.01	0.84	1.23
	Medium	1,981	1,165	58.8	1.13	1.00	1.28
	High	7,223	3,779	52.3	Ref		
Body Mass Index (kg/m^2^)	<18.5	412	180	43.7	1.03	0.81	1.31
	18.5 - <23.0	4,237	2,224	52.5	Ref		
	23.0 - <25.0	2,759	1,565	56.7	0.98	0.88	1.10
	≥25	3,249	1,830	56.3	0.95	0.84	1.06
Cholesterol (mg/dl)	<240	9,228	4,882	52.9	Ref		
	≥240	1,013	624	61.6	1.33	1.14	1.54
Triglyceride (mg/dl)	<150	8,118	4,316	53.2	Ref		
	≥150	2,102	1,179	56.1	0.98	0.87	1.10
Glucose (mg/dl)	<100	7,206	3,786	52.5	Ref		
	100 − <126	2,459	1,378	56.0	0.93	0.84	1.04
	≥126	555	328	59.1	0.98	0.80	1.19
Family history of gastric cancer	No	9,473	5,152	54.4	Ref		
	Yes	1,270	700	55.1	0.96	0.84	1.09

*, **, GI, CI, Ref, same as those of Table 2.

Total subject number of multivariable logistic regression was 8,688.

### Comparison of seroprevalence of *H*. *pylori* among 2011, 2005, and 1998 in asymptomatic subjects without a history of *H*. *pylori* eradication

**Figure 2:**
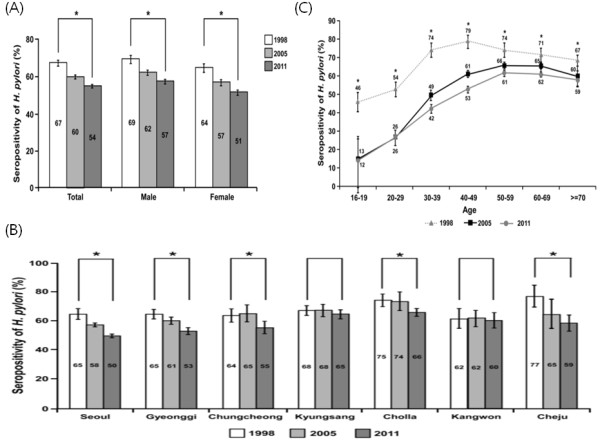
**Trends of seroprevalence of *H***. ***pylori *infection in asymptomatic subjects without a history of *H***. ***pylori *eradication in 1998**[10], **2005**[9], **and 2011.** (^*^p < 0.05) Seroprevalence by sex (Panel **A**), by geographic area (Panel **B**) and by age (Panel **B**).

Comparison of seroprevalence of *H*. *pylori* from 1998, 2005 and 2011 was performed, and the data from 1998 and 2005 were investigated by the Korean *H*. *pylori* Study Group [10] and our group [9], respectively. The overall seroprevalence of *H*. *pylori* infection was 54.4% (95% CI: 53.5-55.4%) in 2011 which is significantly decreased from 66.9 % (95% CI: 65.4-68.6%) in 1998, and 59.6 % (95% CI: 58.5-60.7%) in 2005 (p < 0.001) (Panel A of Figure 2). There was a statistically significant reduction between 1998 and 2005, and between 2005 and 2011.

According to geographic area, the seroprevalence showed a significant downward trend in most of areas over time except in Kyungsang and Kangwon. (Panel B of Figure 2).

The seroprevalence of *H*. *pylori* and 95% CI at intervals of 10 years of age in 1998, 2005, and 2011 were plotted in Panel C of Figure 2. The seroprevalence of *H*. *pylori* was decreased in the all age groups over time with statistical significance from 1998 to 2011. A steep decreasing pattern was observed for subjects under 40 years of age between 1998 and 2005. However, when the time period was extended to 2011, the declining trend was more prominent in older age groups, resulting in an overall decrease for all age groups.

### The birth cohort effects

**Figure 3:**
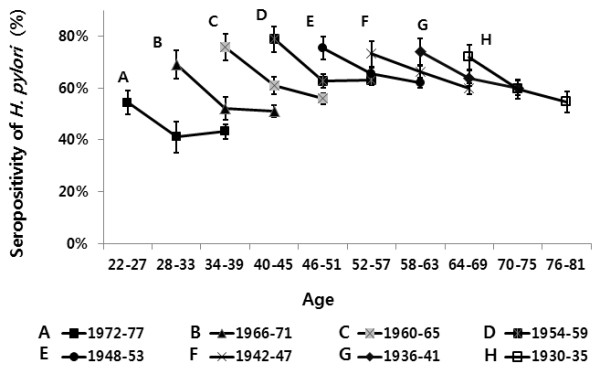
**Seroprevalence of *H*. *pylori *infection in asymptomatic subjects without a history of *H*. *pylori *eradication in birth cohort against age.** Each line connects the values for the same cohort-group in different age group. For example, the first line shows the seroprevalence of *H*. *pylori* in a birth cohort of 1972–77 for ages of 22–39 years, and the second line shows the seroprevalence of *H*. *pylori* in a birth cohort of 1966–71 for ages of 28–45 years. All younger birth cohorts at the same age have a lower seroprevalence of *H*. *pylori* than older birth cohorts.

To observe lifetime trends, the seroprevalence of *H*. *pylori* of categorized birth cohorts against age were plotted as shown in Figure 3. Each line connects the values for the same cohort-group in different age groups. For example, a line first represents a birth cohort of 1972–77 in all graphs. At the same age of 28–33 years (mean 30.5 years old), a younger birth cohort of 1972–77 had a lower seroprevalence of *H*. *pylori* when compared with a older birth cohort of 1966–71. Likewise, all younger birth cohorts at the same age had a lower seroprevalence of *H*. *pylori* compared with older birth cohorts. Within the same birth cohort, most birth cohorts had decreasing pattern of seropositivity of *H*. *pylori* except for a birth cohort of 1972–1977. This birth cohort showed a decreasing pattern from 22–27 to 28–33 years of age, but it showed a very slight increase (from 41% to 43%) from 28–33 to 34–39 years of age.

## Discussion

The decreasing trend (from 66.9% to 54.4%) of seroprevalence of *H*. *pylori* over 13 years was explained by birth cohort analysis, and a relationship between *H*. *pylori* infection and high cholesterol level was found in this large cohort.

A drop in the seroprevalence of *H*. *pylori* infection has been observed in previous studies [2,3,13]. This trend was most often explained by a combination of various factors including rapid economic growth, improved sanitation, and widespread use of antibiotics and proton pump inhibitors [2,3]. Similarly, the overall seroprevalence of *H*. *pylori* significantly decreased in the last survey of the Korean population in 2005 [9] compared with that in 1998 [10], but the declining trend was different depending on the age groups and areas. Although the drop in *H*. *pylori* infection was bigger in younger age groups of subjects 40 years old or less for seven years from 1998 to 2005, the difference of seroprevalence in older groups during same periods was smaller, as shown in the upper two lines of figure 2C. In addition, regarding areas, only subjects who lived in Seoul (capital) and Gyeonggi province which surrounds the capital, showed a clear declining trend during the same periods, but not in all districts in previous study [9]. Furthermore, subjects who lived in Chungcheong province showed a slight increase between 1998 and 2005.

However, when we extended the time period to 2011 in this study, this decreasing trend was more prominent for all ages over 13 years. Similarly, a Japanese study of seropositivity trends of *H*. *pylori* over a period of 10 years from 1992 to 2002–2006 also found declining trends of seropositivity for all age groups [13]. Regarding province, there was no increasing pattern in any province, and a statistically significant decreasing trend was observed in all provinces except two provinces, Kyungsang and Kangwon areas.

We also analyzed birth cohort effects. In the cross sectional study, the prevalence of *H*. *pylori* infection increased till 40 – 49 years of age, after which it remained steady. When we graphically drew the prevalence in *H*. *pylori* infection by birth cohort to differentiate the increase of infection during aging, the seroprevalence was lower in younger birth cohort (i.e. people who were born later) than the older birth cohort (people who were born earlier) at the same age, showing a clear cohort effect in subjects up to 40–45 years of age. This phenomenon could be explained by continuous influx of younger birth cohorts [7]. A similar birth cohort effect for *H*. *pylori* infection was observed in Western studies [2,3,7,8]. In addition, *H*. *pylori* infection in adults is mostly acquired by the age of 15 years [7,8]. One study which followed children (1–3 years old) for 21 years indicated that the annual seroconversion rate had a highest risk at the age of 4–5 years, and newly acquired *H*. *pylori* infections mostly occurred by the age of 10 years [14]. However, there is a doubt whether only a birth cohort effect could explain this pattern. That is, one study in Canada mentioned that an increasing pattern of *H*. *pylori* infection with advancing age may be due to the continuous risk of infection in adults rather than cohort effects [6]. The decrease of *H*. *pylori* seroprevalence with advancing age within the same birth cohort in our study strongly suggests that aging is not likely to raise risk of *H*. *pylori* infection. There was a decreasing effect with advancing age within the same birth cohorts. This might have occurred as a result of cases taking antibiotics or proton pump inhibitors even without formal eradication therapy of *H*. *pylori*[15].

There have been several studies regarding risk factors of *H*. *pylori* infection [9,14,16-18], but their results are still unclear, except socioeconomic status as the risk factors. Our results also showed that lower social economic status is associated with the risk of *H*. *pylori* infection in a cross sectional analysis. Furthermore, subjects with lower social economic status had a lower likelihood of taking *H*. *pylori* eradication therapy in the present study. Interestingly, our study showed a relationship between cholesterol level and seropositivity of *H*. *pylori*. Subjects who had a TC level of ≥240 mg/dl were 1.3 times more likely to be seropositive for *H*. *pylori*. In frequency analysis, higher levels of TG and glucose as well as TC were also associated with *H*. *pylori* infection, but after adjusting for demographic variables, clinical information, and socioeconomic status(i.e. age, BMI, income and etc.), only TC among metabolic parameters was related to *H*. *pylori* infection. So far, the results regarding the relationship between lipid parameters such as TC, TG and low-density lipoprotein cholesterol (LDL-C) levels and *H*. *pylori* seropositivity have not been consistent. Some studies [19-21] reported no relationship, but several studies reported higher atherogenic lipid parameter levels in *H*. *pylori* seropositive subjects in comparison with seronegative ones [22-24] as seen in the present study. Our study results could be convincing for demonstrating the effect of *H*. *pylori* infection on atherosclerotic disease because the positive relationship between TC and *H*. *pylori* seropositivity was persistent even after adjustment for BMI and age in a large cohort. The mechanism of how *H*. *pylori* infection modifies the serum lipid profiles is still not clear, but a plausible explanation is that systemic inflammatory response to the bacterium induces changes in lipid and lipoprotein metabolism [25]. That is, chronic *H*. *pylori* infection has been postulated to shift the lipid profile toward an atherogenic direction *via* the action of proinflammatory cytokines, such as interleukins 1 and 6, interferon-alpha, and tumor necrosis factor-alpha. These cytokines are capable of affecting lipid metabolism in various ways, including activation of adipose tissue lipoprotein lipase, stimulation of hepatic fatty acid synthesis, influencing lipolysis and the increasing hepatic HMG-CoA reductase activity [26,27]. Thus, *H*. *pylori* infection could play a role in the atherosclerotic process and may be a reliable indicator for the assessment of cardiovascular disease risk.

There are several limitations which should be acknowledged in this study. First, the relationship between *H*. *pylori* infection and its risk factors in the cross sectional study could not be proven conclusively. However, this is an unavoidable limitation in the cross sectional study. Second, we compared the time trends of seroprevalence of *H*. *pylori* using two previous studies [9,10]. However, the responsible author (N.K.) did play main role in these previous studies, and the population in 2011 study was restricted to have comparability of *H*. *pylori* seroprevalence. In other words, the subjects in 2011 study were restricted to asymptomatic people without a history of *H*. *pylori* eradication and GI operation. Moreover, this study was carried out nationwide, so our findings represent a national trend, not a local phenomenon. Nonetheless, the study subjects in 1998 involved a relatively lower population from Seoul and Gyeonggi, (capital city and its near city) compared with the population in 2005 and 2011. Generally people in capital cities have higher socioeconomic conditions than those living in other areas. It may account for much higher seroprevalence in 1998 compared with 2005/2011. However, the change of seroprevalence by the strata (e.g. age, sex, region, etc.) over time periods may indicate that our overall result is not much influenced by a different proportion of subjects from provinces. Third, for the generation of synthetic cohort, cross-sectional data should have the same interval. However, our data did not have the same interval as the previous data. This is the reason why we considered the data from 1998 as equivalent to those from 1999. This intentional modification could have caused bias, but we think that the bias may be negligible because *H*.*pylori* seroprevalence was not changed much by one-year.

## Conclusion

In conclusion, we confirmed that the seropositivity of *H*. *pylori* declined across all age groups from 1998 to 2011 using nationwide data, an effect which originated from birth cohort effects and continuous risk reduction of *H*. *pylori* infection during one’s life time. In addition, we found that high TC level as well as lower social economic status had a relationship with *H*. *pylori* infection. These results may suggest the importance of management of *H*. *pylori* infection in younger age and the effect of *H*. *pylori* infection on atherosclerosis.

## Abbreviations

GC: Gastric cancer; GI: Gastrointestinal; H. pylori: Helicobacter pylori; TG: Triglyceride; TC: Total cholesterol.

## Competing interests

All authors declare that they have no conflict of interest.

## Authors’ contributions

SHL carried out the acquisition of data, analysis and interpretation of data, and drafting of the manuscript; JK carried out statistical analysis, interpretation of data, and drafting of the manuscript; NK carried out study concept and design, critical revision of the manuscript for important intellectual content and study supervision as a corresponder; GHK participated in acquisition of the data of southeastern part of Korea; JMK participated in design of the study and acquisition of data; MJP participated in acquisition of data; JYY participated in acquisition of data and study concept; HUK participated in acquisition of the data of southernmost part of Korea; GHB participated in acquisition of the data of northeastern part of Korea; GSS participated in acquisition of the data of western part of Korea; JES participated in acquisition of the data of middle upcountry of Korea; YEJ participated in acquisition of the data of southwestern part of Korea ;JSK participated in technical or material support and study supervision ;HCJ participated in study supervision and provided general support. All authors read and approved the final manuscript.

## Pre-publication history

The pre-publication history for this paper can be accessed here:

http://www.biomedcentral.com/1471-230X/13/104/prepub

## References

[B1] Egan BJ Holmes K O'Connor HJ O'Morain CA Helicobacter pylori gastritis, the unifying concept for gastric diseases Helicobacter 2007 12 Suppl 2 39 44 17991175 10.1111/j.1523-5378.2007.00575.x

[B2] Parsonnet J The incidence of Helicobacter pylori infection Aliment Pharmacol Ther 1995 9 Suppl 2 45 51 8547528

[B3] Roosendaal R Kuipers EJ Buitenwerf J Meuwissen SG van Kamp GJ Vandenbroucke-Grauls CM Helicobacter pylori and the birth cohort effect: evidence of a continuous decrease of infection rates in childhood Am J Gastroenterol 1997 92 1480 1482 9317067

[B4] Taylor DN Blaser MJ The epidemiology of Helicobacter pylori infection Epidemiol Rev 1991 13 42 59 1765119 10.1093/oxfordjournals.epirev.a036078

[B5] Graham DY Malaty HM Evans DG Evans DJ Jr Klein PD Adam E Epidemiology of Helicobacter pylori in an asymptomatic population in the United States. Effect of age, race, and socioeconomic status Gastroenterology 1991 100 1495 1501 2019355 10.1016/0016-5085(91)90644-z

[B6] van Zanten SJ V Pollak PT Best LM Bezanson GS Marrie T Increasing prevalence of Helicobacter pylori infection with age: continuous risk of infection in adults rather than cohort effect J Infect Dis 1994 169 434 437 8106778 10.1093/infdis/169.2.434

[B7] Banatvala N Mayo K Megraud F Jennings R Deeks JJ Feldman RA The cohort effect and Helicobacter pylori J Infect Dis 1993 168 219 221 8515114 10.1093/infdis/168.1.219

[B8] Kosunen TU Aromaa A Knekt P Salomaa A Rautelin H Lohi P Heinonen OP Helicobacter antibodies in 1973 and 1994 in the adult population of Vammala, Finland Epidemiol Infect 1997 119 29 34 9287940 10.1017/s0950268897007565 PMC2808819

[B9] Yim JY Kim N Choi SH Kim YS Cho KR Kim SS Seo KS Kim HU Baik GH Sin CS Cho SH Oh BH Seroprevalence of Helicobacter pylori in South Korea Helicobacter 2007 12 333 340 17669107 10.1111/j.1523-5378.2007.00504.x

[B10] Kim JH Kim HY Kim N Kim SW Kim JG Kim JJ Roe IH SJK Sim JG Ahn H Yoon BC Lee SW Lee YC Chung IS Jung HY Hong WS Choi KW Seroepidemiological study of Helicobacter pylori infection in asymptomatic people in South Korea J Gastroenterol Hepatol 2001 16 969 975 11595059 10.1046/j.1440-1746.2001.02568.x

[B11] Kim SY Ahn JS Ha YJ Doh HJ Jang MH Chung SI Park HJ Serodiagnosis of Helicobacter pylori infection in Korean patients using enzyme-linked immunosorbent assay J Immunoassay 1998 19 251 270 9840297 10.1080/01971529808005485

[B12] Kwon JW Song YM Sung J Sohn Y Cho SI Varying patterns of BMI increase in sex and birth cohorts of Korean adults Obesity (Silver Spring) 2007 15 277 282 17299099 10.1038/oby.2007.539

[B13] Shiota S Murakami K Fujioka T Yamaoka Y Population-based strategies for Helicobacter pylori-associated disease management: a Japanese perspective Expert Rev Gastroenterol Hepatol 2010 4 149 156 20350262 10.1586/egh.10.7 PMC2896743

[B14] Malaty HM El-Kasabany A Graham DY Miller CC Reddy SG Srinivasan SR Yamaoka Y Berenson GS Age at acquisition of Helicobacter pylori infection: a follow-up study from infancy to adulthood Lancet 2002 359 931 935 11918912 10.1016/S0140-6736(02)08025-X

[B15] Kim N Lim SH Lee KH Kim JM Cho SI Jung HC Song IS Seroconversion of Helicobacter pylori in Korean male employees Scand J Gastroenterol 2005 40 1021 1027 16211701 10.1080/00365520510015917

[B16] Perez-Perez GI Rothenbacher D Brenner H Epidemiology of Helicobacter pylori infection Helicobacter 2004 9 Suppl 1 1 6 15347299 10.1111/j.1083-4389.2004.00248.x

[B17] The EUROGAST Study Group Epidemiology of, and risk factors for, Helicobacter pylori infection among 3194 asymptomatic subjects in 17 populations Gut 1993 34 1672 1676 8282253 10.1136/gut.34.12.1672 PMC1374460

[B18] Moayyedi P Axon AT Feltbower R Duffett S Crocombe W Braunholtz D Richards IDG Dowell AC Forman D Relation of adult lifestyle and socioeconomic factors to the prevalence of Helicobacter pylori infection Int J Epidemiol 2002 31 624 631 12055165 10.1093/ije/31.3.624

[B19] Danesh J Peto R Risk factors for coronary heart disease and infection with Helicobacter pylori: meta-analysis of 18 studies BMJ 1998 316 1130 1132 9552950 10.1136/bmj.316.7138.1130 PMC28515

[B20] Oshima T Ozono R Yano Y Oishi Y Teragawa H Higashi Y Yoshizumi M Kambe M Association of Helicobacter pylori infection with systemic inflammation and endothelial dysfunction in healthy male subjects J Am Coll Cardiol 2005 45 1219 1222 15837252 10.1016/j.jacc.2005.01.019

[B21] Paximadas S Pagoni ST Kosmidis M Tsarouchas X Christou M Chatziantonakis N Papachilleos P The lipid profile in adults with negative or positive antibody Helicobacter pylori [abstract] Atherosclerosis supplement 2005 6 74

[B22] Kucukazman M Yavuz B Sacikara M Asilturk Z Ata N Ertugrul DT Yalcin AA Yenigum EC Kizilca G Okten H Akin KO Nazligul Y The relationship between updated Sydney System score and LDL cholesterol levels in patients infected with Helicobacter pylori Dig Dis Sci 2009 54 604 607 18649137 10.1007/s10620-008-0391-y

[B23] Laurila A Bloigu A Nayha S Hassi H Leinonen M Saikku P Association of Helicobacter pylori infection with elevated serum lipids Atherosclerosis 1999 142 207 210 9920523 10.1016/s0021-9150(98)00194-4

[B24] Majka J Rog T Konturek PC Konturek SJ Bielanski W Kowalsky M Szczudlik A Influence of chronic Helicobacter pylori infection on ischemic cerebral stroke risk factors Med Sci Moni 2002 8 CR675 CR684 12388919

[B25] Gallin JI Kaye D O'Leary WM Serum lipids in infection N Engl J Med 1969 281 1081 1086 5824173 10.1056/NEJM196911132812001

[B26] Grunfeld C Gulli R Moser AH Gavin LA Feingold KR Effect of tumor necrosis factor administration in vivo on lipoprotein lipase activity in various tissues of the rat J Lipid Res 1989 30 579 585 2754338

[B27] Memon RA Grunfeld C Moser AH Feingold KR Tumor necrosis factor mediates the effects of endotoxin on cholesterol and triglyceride metabolism in mice Endocrinology 1993 132 2246 2253 8477669 10.1210/endo.132.5.8477669

